# Functional brain connectivity changes associated with day-to-day fluctuations in affective states

**DOI:** 10.3758/s13415-024-01216-6

**Published:** 2024-09-25

**Authors:** Jeanne Racicot, Salima Smine, Kamran Afzali, Pierre Orban

**Affiliations:** 1 Centre de Recherche de l’Institut Universitaire en Santé Mentale de Montréal, Montréal, Canada; 2 Département de Psychiatrie et d’addictologie, Université de Montréal, Montréal, Canada; 3 Consortium Santé Numérique, Université de Montréal, Montréal, Canada

**Keywords:** Amygdala, Emotion, Functional connectivity, Mood, Prefrontal cortex

## Abstract

Affective neuroscience has traditionally relied on cross-sectional studies to uncover the brain correlates of affects, emotions, and moods. Such findings obfuscate intraindividual variability that may reveal meaningful changing affect states. The few functional magnetic resonance imaging longitudinal studies that have linked changes in brain function to the ebbs and flows of affective states over time have mostly investigated a single individual. In this study, we explored how the functional connectivity of brain areas associated with affective processes can explain within-person fluctuations in self-reported positive and negative affects across several subjects. To do so, we leveraged the Day2day dataset that includes 40 to 50 resting-state functional magnetic resonance imaging scans along self-reported positive and negative affectivity from a sample of six healthy participants. Sparse multivariate mixed-effect linear models could explain 15% and 11% of the within-person variation in positive and negative affective states, respectively. Evaluation of these models’ generalizability to new data demonstrated the ability to predict approximately 5% and 2% of positive and negative affect variation. The functional connectivity of limbic areas, such as the amygdala, hippocampus, and insula, appeared most important to explain the temporal dynamics of affects over days, weeks, and months.

## Introduction

Affective processes, encompassing core affects, emotions, and moods (Ekkekakis, 2013), play a crucial role in everyday life, with their dysregulation being central in various psychopathologies (Marroquín et al., 2017; Stanton & Watson, 2014; Yardley & Rice, 1991). Functional magnetic resonance imaging (fMRI) in humans has contributed to characterize the neural correlates of affective processes. Meta-analyses of activation studies using tasks that elicit specific categories of affective components support the idea that distinct affect and emotions are not localized in dedicated brain regions or networks, but rather involve the interplay of a distributed set of domain-general brain areas (Gündem et al., 2022; Kober et al., 2008; Lindquist et al., 2012, 2016; Riedel et al., 2018; Vytal & Hamann, 2010; Wager et al., 2015). Commonly associated with affective processes are limbic regions, such as the amygdala, hippocampus, insula, ventral striatum, and anterior cingulate cortex, alongside various areas of lateral and medial prefrontal cortices. Additional implicated regions include the temporal lobe (e.g., the fusiform gyrus), the supplementary motor area, and parts of the cerebellum. Hemispheric asymmetries in the recruitment of these regions have been reported in functional brain imaging studies, although there is no single consensus on hemispheric preferences for positive versus negative affectivity (Kober et al., 2008; Lindquist et al., 2012, 2016; Riedel et al., 2018; Rohr et al., 2013; Vytal & Hamann, 2010; Wager et al., 2015). Beyond task-based activation studies, research has exploried the modulation of functional brain connectivity by affective processes using resting-state fMRI, which is not susceptible to practice effects in the context of repeated measures, among other benefits. Resting-state fMRI studies has revealed associations between affective processes and various intrinsic networks encompassing the aforementioned brain regions, including the default-mode, salience, and visual networks (Eryilmaz et al., 2011; Harrison et al., 2008; Rohr et al., 2013; Satpute & Lindquist, 2019; Touroutoglou et al., 2015; Underwood et al., 2021). For instance, the default-mode network has been hypothesized to represent the internal experience of emotions and to facilitate the discrimination between affective states (Eryilmaz et al., 2011; Saarimäki et al., 2022; Satpute & Lindquist, 2019).

Findings from affective neuroscience have predominantly relied on cross-sectional study designs utilizing group averages, where individual data at a single time point are amalgamated into an idealized average subject. However, such reliance on group averages obfuscate meaningful variability at both the inter- and intra-individual levels (Chen et al., 2021; Fisher et al., 2018; Hamaker, 2012; Kraus et al., 2023; Michon et al., 2022; Molenaar & Campbell, 2009; Naselaris et al., 2021; Seghier & Price, 2018; Van Horn et al., 2008).The interpretation of findings based on group averages often fall prey to ecological fallacies, leading to erroneous inferences about individuals within the group and overlooking the true complexity of the brain-behavior relationships (Fisher et al., 2018; Hamaker, 2012; Molenaar & Campbell, 2009; Van Horn et al., 2008). It is possible to obtain reliable results at group level using data that is unreliable across time in the subjects that define the group (Fröhner et al., 2019). Therefore, brain imaging studies that focus on unraveling inter- and intra-individual variability are necessary to properly characterize stable traits and transient states, respectively (Chen et al., 2021; Cornblath et al., 2019; Kraus et al., 2023; Lynch et al., 2023; Treadway & Leonard, 2016; Yarkoni & Braver, 2010).

There has been growing interest in the characterization of inter-individual traits with precision neuroimaging studies as of late (Fedorenko, 2021; Kraus et al., 2023; Marek et al., 2022; Michon et al., 2022; Seghier & Price, 2018). However, as noted by Molenaar and Campbell (2009) and demonstrated by Fisher et al. (2018), trait-level effects cannot be directly applied to state-level processes without prior validation. Equivalence between inter- and intra-individual variabilities would require the studied processes to be ergodic—homogenous in a population and static across time. This condition, notably, does not hold true for most if not all processes in cognitive and psychiatric neuroscience (Fisher et al., 2018; Hamaker, 2012; Molenaar & Campbell, 2009). While functional brain organization exhibits most variability at the inter-individual level (Gratton et al., 2018), notable dynamic processes occur at the intra-individual level, manifesting at various temporal resolutions, spanning minutes, hours, days, or weeks. These temporal dynamics in brain function likely reflect in part relevant cognitive and affective processes. The study of intra-individual variability in brain function holds particular relevance in psychopathology where periods of remission alternate with recurrent relapses. This approach holds the potential to identify optimal treatment timing or pinpoint when a treatment modification is necessary (Cornblath et al., 2019; Heller, 2016; Scangos et al., 2021). Indeed, promising biomarkers for psychiatric disorders, such as activations of amygdala and the cingulate cortex in response to emotional tasks for the treatment of mood disorders, exhibit significant between-session variability at the intra-individual level (Nord et al., 2017).

Intraindividual changes in human brain function have been observed at various temporal scales, in relation to multiple physical, cognitive, and behavioral variables. Brain activation levels can elucidate fluctuations in behavioral performance at the single trial level, presumably as a function of attention (Pessoa et al., 2002; Ress et al., 2000; Sapir et al., 2005). These attention-related effects exhibit robustness across diverse temporal resolutions (Rosenberg et al., 2020). The unprecedented accumulation of extensive resting-state fMRI data, coupled with various phenotypic variables in a single subject over 18 months (Laumann et al., 2015; Poldrack et al., 2015), has enabled the exploration of the relationship between functional brain connectivity and attention levels over days, weeks, and months (Shine et al., 2016). Additional resting-state functional brain connectivity studies have revealed intra-individual variations associated with the content of thoughts (Brennan et al., 2021), hormone levels (Mueller et al., 2021; Pritschet et al., 2020, 2021), the circadian rhythm (Hodkinson et al., 2014; Shannon et al., 2013), and the occurrence of migraines (Filippi et al., 2022; Stankewitz & Schulz, 2022). Within the affective domain, the intensively repetitive scanning of a healthy subject over months (Laumann et al., 2015; Poldrack et al., 2015) has revealed an increase in network flexibility in positive mood (Betzel et al., 2017). Positive mood was further associated with the functional connectivity of the subgenual anterior cingulate cortex (Mirchi et al., 2018). Task-based fMRI activation studies of basic affective dimensions (valence and arousal) have reported intra-individual associations in frontal, limbic, visual, and temporal regions (Baucom et al., 2012; Bush et al., 2018; Kim et al., 2016). Using intracranial electroencephalography, fluctuations in self-reported moods over several days could be predicted in a majority of subjects using amygdala-hippocampus coherence (Kirkby et al., 2018) and other limbic regions, such as the orbitofrontal cortex (Sani et al., 2018). In clinical populations, intensive longitudinal resting-state fMRI studies have linked amygdala connectivity with distinct mood states (euthymia, depression, mania/hypomania) in bipolar disorders (Rey et al., 2021) as well as frontostriatal connectivity with anhedonia symptoms in individuals with depression (Lynch et al., 2023).

In the present study, we further explored how functional connectivity of brain regions previously associated with affective processes could explain within-person fluctuating moods over weeks and months at the group level in several healthy subjects. In contrast to previous studies that focussed on a single subject, our goal was thus to study intra-individual associations that were commonly observed in a group of individuals. We leveraged the Day2day dataset, which includes resting-state fMRI data as well as self-reported positive and negative affects in a small group of healthy subjects scanned on numerous occasions over approximately half a year (Filevich et al., 2017). This intensively longitudinal brain imaging dataset was previously utilized to investigate intra-individual changes in brain function related to time spent outdoors (Kühn et al., 2021), changes in weather (Di et al., 2022), as well as several physiological, metabolic, hormonal, and environmental factors (Karch et al., 2019). Using multivariate mixed-effect linear modeling, we assessed the extent to which functional brain connectivity can explain within-person variability in positive and negative affectivity across subjects. Additionally, we evaluated the models’ ability to generalize and accurately predict previously unseen self-reported moods in these subjects (Rosenberg & Finn, 2022; Yarkoni & Westfall, 2017). Because of the limited number of subjects, our analyses thus emphasized within-person effects averaged across subjects, without characterizing between-person effects.

## Methods

### Participants

The Day2day dataset (Filevich et al., 2017; Kühn et al., 2021) comprises intensively longitudinal brain imaging and behavioral data from a cohort of six young (age 24–32 years), healthy (free of medication and psychiatric illness) individuals (5 females, 1 male). Participants underwent functional magnetic resonance imaging (fMRI) across 40 to 50 sessions irregularly distributed over a span of 6 to 8 months. These participants were employed at the Max Planck Institute for Human Development, where data were acquired between July 2013 and February 2014. The study received approval from the Ethics Committee of Charité University Clinic in Berlin, Germany, and was conducted in accordance with the Declaration of Helsinki.

### Behavioral measures

Repeated assessments of mood were conducted using the Positive and Negative Affect Schedule (PANAS) developed by Watson et al. (1988). The PANAS is a self-report questionnaire comprising 20 mood terms, 10 of which are associated with positive affectivity and 10 others reflecting negative affectivity (Table 1). Participants rated the extent to which they felt each mood term on a 5-point Likert scale: 1 = very slightly or not at all; 2 = a little; 3 = moderately; 4 = quite a bit; 5 = extremely. They were instructed to report affectivity at the time of scanning (Filevich et al., 2017). The positive and negative scales were designed to be orthogonal, representing states of high-arousal positivity and high-arousal negativity. Lower scores of each dimension are indicative of states of calm (Barrett & Russell, 1999; Watson et al., 1988). Positive and negative affectivity measures demonstrate the separability of these dimensions at the intra-individual level (Watson et al., 1988, 1999; Zevon & Tellegen, 1982).

**Table 1:** PANAS. Compilation of the 20 PANAS items categorized into the positive or negative affect scale according to the classification by Watson et al. (1988)

Positive affect	Negative affect
Active	Afraid
Alert	Ashamed
Attentive	Distressed
Determined	Guilty
Enthusiastic	Hostile
Excited	Irritable
Inspired	Jittery
Interested	Nervous
Proud	Scared
Strong	Upset

### Brain imaging

#### Data acquisition

Brain images were collected on a 3 T Magnetom Trio MRI scanner system (Siemens Medical Systems, Erlangen, Germany), using a 12-channel radiofrequency head coil. T1*-weighted structural images were collected using a three-dimensional T1-weighted magnetization prepared gradient-echo sequence (MPRAGE) using the following parameters: TR = 2500 ms, TE = 4.77 ms, TI = 1100 ms, FOV = 256 × 256 × 192 mm^3^, flip angle = 7°, bandwidth = 140 Hz/pixel, voxel size = 1 × 1 × 1 mm^3^, duration = 9:20 min. T2*-weighted echo planar imaging (EPI) sequence sensitive to blood oxygen level dependent (BOLD) contrast was used to collect resting-state functional images using the following parameters: TR = 2000 ms, TE = 30 ms, FOV = 216 × 216 × 129 mm^3^, flip angle = 80°, bandwidth = 2042 Hz/pixel, voxel size = 3 × 3 × 3 mm^3^, distance factor = 20%, 36 axial slices using GRAPPA acceleration factor 2, duration = 5:08 min. Subjects kept their eyes closed throughout all brain imaging data acquisitions.

#### Spatial preprocessing

Brain images were preprocessed with fMRIPrep (Esteban et al., 2019; Esteban, Markiewicz et al., 2020), which is based on Nipype (Esteban Markiewicz et al., 2020; Gorgolewski et al., 2011), using default settings. Structural (T1*-weighted) images were skull-stripped, and tissues were segmented (cerebrospinal fluid, white, and gray matters). Volume-based spatial normalization into the MNI152 space was conducted by using nonlinear registration. Functional (T2*-weighted) BOLD images were co-registered with the anatomical image separately for each session. Head motions were estimated with six rotation and translation parameters. After high-pass filtering, head motions, confounds derived from cerebrospinal fluid and white matter, as well as six noise components extracted using anatomical CompCor (Behzadi et al., 2007) were regressed out from the fMRIPrep output (Wang et al., 2023). The CompCor method was used to account for the global signal and motion artefacts; a low-parameter approach was used given the young, healthy subjects contributed brain imaging data with minimal motion (Ciric et al., 2017; Satterthwaite et al., 2019). Indeed, framewise displacement (FD) at the subject level was very low; no images exceeded the threshold of > 0.5 mm. The mean and standard deviation values of FD (mm) are as follows: Subject 1 = 0.10 ± 0.02; Subject 3 = 0.12 ± 0.04; Subject 5 = 0.13 ± 0.06; Subject 6 = 0.09 ± 0.03; Subject 7 = 0.11 ± 0.03; Subject 8 = 0.10 ± 0.01. Spatial smoothing was omitted to retain independence between selected regions, ensuring the integrity of the functional connectivity measures (Alakörkkö et al., 2017).

#### Regions of interest

**Fig. 1:**
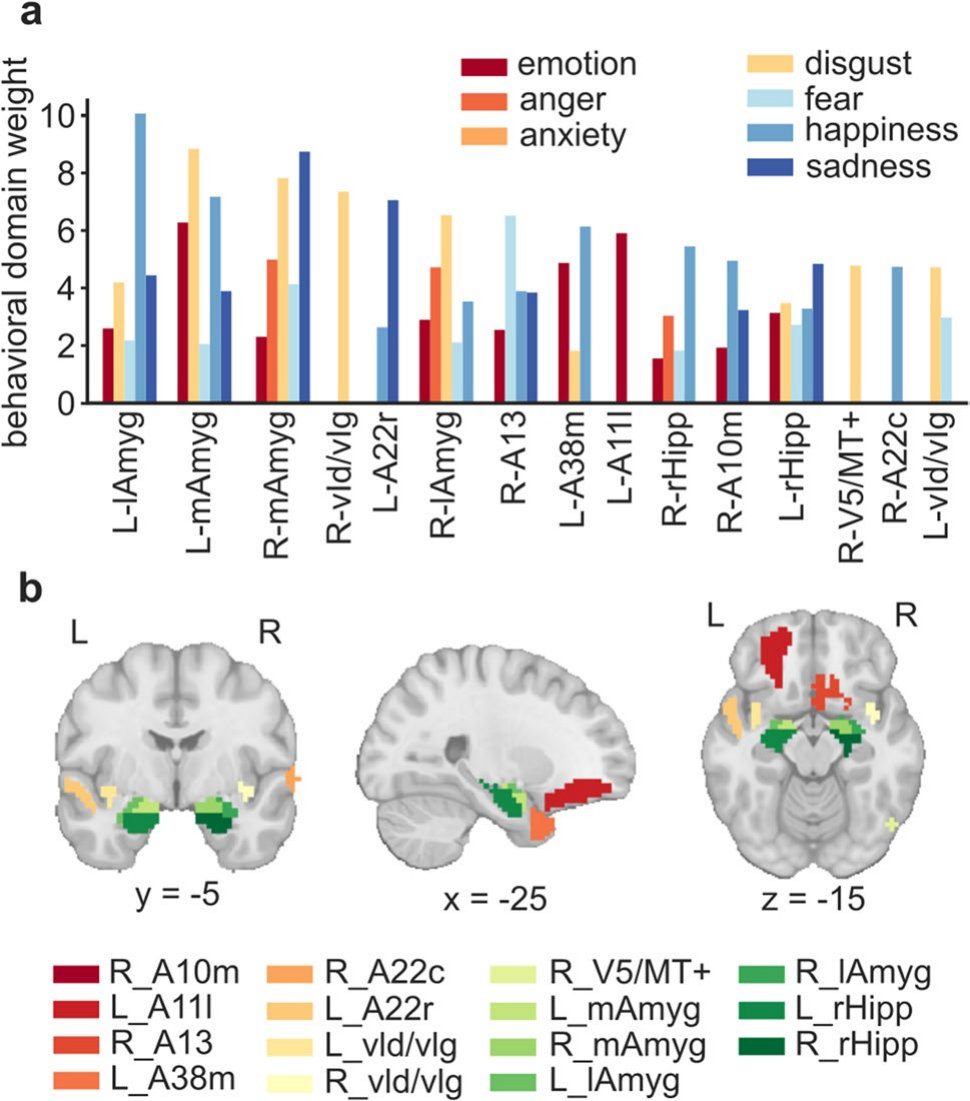
Fifteen regions of interest (ROIs) selected from the Human Brainnetome Atlas (Fan et al., 2016) based on their documented involvement in affective processes. (**a**) ROIs were chosen based on their highest scores associated with the “Emotion” behavioral domain or subdomain (anger, anxiety, disgust, fear, happiness, sadness) as defined in the BrainMap taxonomy (Fox & Lancaster, 2002; Fox et al., 2005). (**b**) Visualization of all ROIs in the MNI space, excluding R_A10

Our analysis centered on functional connectivity among a set of fine-grained brain regions previously associated with affective processes in the literature. These regions of interest (ROIs) were selected from the Human Brainnetome Atlas (Fan et al., 2016), which comprises 210 cortical and 36 subcortical nonoverlapping brain regions based on structural and functional connectivity measures. The 246 brain regions of this atlas were mapped to mental processes by reference to the BrainMap taxonomy (Fox & Lancaster, 2002; Fox et al., 2005). We isolated the 15 brain regions with the highest scores associated with the “Emotion” behavioral domain and subdomains (anger, anxiety, disgust, fear, happiness, sadness), which reflect the mental operations likely linked to activation in those brain regions based on forward and reverse inferences. The 15 ROIs were found in the amygdala, hippocampus, and insula as well as in frontal, temporal, and occipital cortices (Fig. 1; Table 2).

**Table 2:** Regions of interest. Fifteen ROIs selected from the Human Brainnetome Atlas (Fan et al., 2016), featuring their cyto-architectonic designation alongside the abbreviations employed in this study. Certain brain areas were only selected from either the left (L) or right (R) hemisphere. MNI stereotactic coordinates indicate the center of mass of an ROI. The codes and numbers assigned to ROIs in the original atlas paper also are provided

Modified cyto-architectonic	Abbreviation	Hemisphere	Gyrus	MNI (x,y,z)	Area code	Number
Medial area 10	A10m	R	Superior frontal gyrus	8, 58, 13	SFG_R_7_7	14
Lateral area 11	A11l	L	Orbital gyrus	-23, 38, -18	OrG_L _6_3	45
Area 13	A13	R	Orbital gyrus	9, 20, -19	OrG_R_6_5	50
Medial area 38	A38m	L	Superior temporal gyrus	-32, 14, -34	STG_L_6_1	69
Caudal area 22	A22c	R	Superior temporal gyrus	66, -20, 6	STG_R_6_4	76
Rostral area 22	A22r	L	Superior temporal gyrus	-55, -3, -10	STG_L_6_6	79
Ventral dysgranular and granular insula	vId/vIg	L R	Insular lobe	-38, -4, -9 39, -2, -9	INS_L_6_4 INS_R_6_4	169 170
Area V5/MT +	V5/MT +	R	Lateral occipital cortex	48, -70, -1	LOcC_R_4_2	202
Medial amygdala	mAmyg	L R	Amygdala	-19, -2, -20 19, -2, -19	Amyg_R_2_1 Amyg_L_2_1	211 212
Lateral amygdala	lAmyg	L R	Amygdala	-27, -4, -20 28, -3, -20	Amyg_L_2_2 Amyg_R_2_2	213 214
Rostral hippocampus	rHipp	L R	Hippocampus	-22, -14, -19 22, -12, -20	Hipp_L_2_1 Hipp_R_2_1	215 216

#### Functional brain connectivity

Pairwise functional connectivity measures between brain ROIs were extracted following the application of a low-pass filter of 0.01. Pearson correlation coefficients were computed and z-transformed with Nilearn (Nilearn contributors et al., 2023) for all ROIs pairs, resulting in 105 unique connectivity measures, which were considered in mass univariate statistical analyses. Given the high dimensionality of such connectivity data in comparison to the total number of sessions, we applied dimensionality reduction through principal component analysis (PCA) (Jolliffe, 2002; Pearson, 1901) using Scikit-Learn (Pedregosa et al., 2011). The 30 coupling components, jointly explaining > 80% of the variance, served as predictors in multivariate statistical analyses.

### Statistical modeling

Multivariate mixed-effect linear modeling (McLean et al., 1991) was first applied to explain affectivity levels, separately for positive and negative affectivity, using all functional brain connectivity measures (i.e., 30 coupling components). Person-mean centered connectivity measures over multiple sessions were modeled as time-varying predictors to characterize within-person fluctuations of connectivity measures. The number of days since the first scan was entered as confounding covariate, and a random intercept term was specified to account for between-person variability in the outcome measures. Because of the small sample size and a large number of parameters, Least Absolute Shrinkage and Selection Operator (LASSO) regularization (Tibshirani, 1996) was applied. Based on a threshold hyperparameter, magnitude of the coefficients, and a sample size dependent penalty parameter, models shrank small coefficients to zero and estimated the coefficients with higher relative importance. The coefficient of determination R^2^ quantified the proportion (%) of the within-person variability of each affectivity outcome that could be explained by all connectivity measures taken altogether. In addition to using LASSO regularization highlighting certain coupling components, we performed permutation feature importance to assess the loss in R^2^ that resulted from shuffling the values of a given component. We further assessed to what extent affectivity levels could be predicted rather than explained by connectivity measures (Yarkoni & Westfall, 2017) through testing the fitted models on data unseen during model training using fivefold longitudinal cross-validation (Grimm et al., 2017). Each subject was proportionally represented across training and test subsets. Mass univariate mixed-effect linear modeling was also conducted by estimating separate models for each one of the 105 raw connectivity measures. The resulting *p*-values were corrected for multiple comparisons by using a false discovery rate (FDR) procedure (Benjamini & Yekutieli, 2001). Statistical analyses were conducted by using the *glmmPLQ* function of the *MASS* package (https://cran.r-project.org/web/packages/MASS/index.html), the *glmmLasso* package (https://cran.r-project.org/web/packages/glmmLasso/index.html), and the *lmer* function of the *lme4* package (https://cran.r-project.org/web/packages/lme4/index.html) in the statistical environment R (R Core Team, 2023).

## Results

### Variability in affectivity

**Fig. 2:**
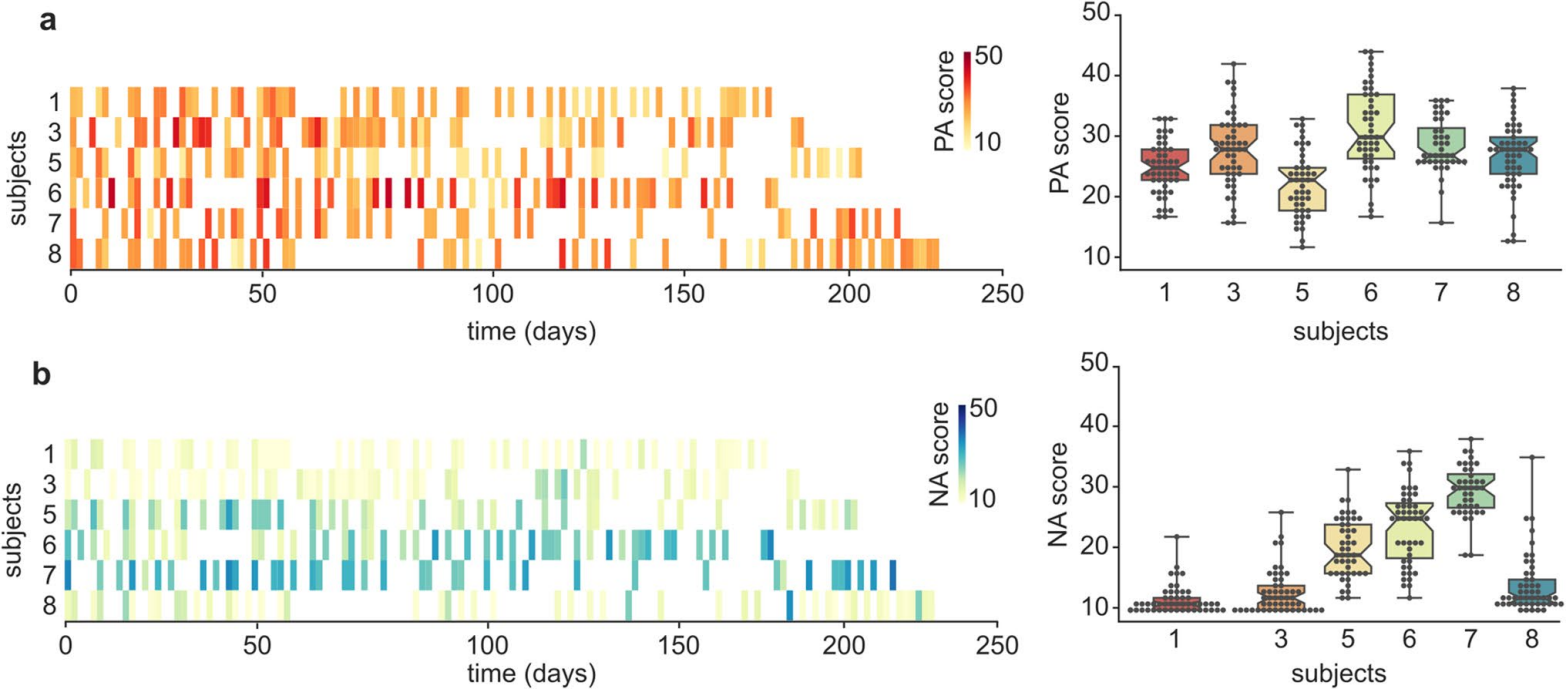
Distribution of Positive and Negative Affect scores. Fluctuations in positive affectivity (PA) (**a**) and negative affectivity (NA) (**b**) scores are presented across 40 to 50 longitudinal sessions for each subject, distributed over 6 to 8 months (left). Boxplots and swarmplots (right) portray inter- and intra-individual variability. Subject numbering corresponds to that found in the data paper describing the Day2day dataset (Filevich et al., 2017)

Positive and negative affect scores, derived from the summation of their respective items, exhibited significant variation at both the inter- and intra-individual levels in the Day2day sample (Fig. 2; Table 3). Substantial between-person differences were observed in the average levels of affectivity, both in the positive (mean ± SD = 26.98 ± 3.03; F(5,275) = 13.88, *p* < 0.001) and negative (mean ± SD = 18.68 ± 7.03; F(5,275) = 111.78, *p* < 0.001) domains. Within-person variation was more pronounced for positive affectivity, as indicated by a 1.8/1 ratio of intra-individual over inter-individual dispersion (SD). Conversely, a reverse effect was seen in the case of negative affectivity, with a 0.61/1 ratio, although excluding the three subjects presenting noticeable floor effects (subjects 1, 3, and 8) revealed approximately equal levels of inter- and intra-individual variability (ratio = 1.1/1).

**Table 3:** Variability in affectivity. For each subject, numbered as in the original article by Filevich et al. (2017), this table provides information on the number of sessions, duration of the protocol, and descriptive statistics related to positive and negative affect scores

Subject	No. sessions	Total no. days (mean interval between sessions)	Positive affectivity Mean ± SD (range)	Negative affectivity Mean ± SD (range)
1	50	168 (3.4)	25.0 ± 4.18 (17–33)	11.7 ± 2.34 (10–22)
3	50	189 (3.8)	27.88 ± 6 (16–42)	12.92 ± 3.6 (10–26)
5	45	208 (4.6)	22.4 ± 5.32 (12–33)	20.11 ± 4.88 (12–33)
6	47	170 (3.6)	31.17 ± 6.95 (17–44)	23.72 ± 6.0 (12–36)
7	40	218 (5.5)	28.6 ± 4.4 (16–36)	29.5 ± 4.16 (19–38)
8	49	232 (4.7)	26.8 ± 5.64 (13–38)	14.12 ± 4.88 (10–35)

### Functional brain connectivity

**Fig. 3:**
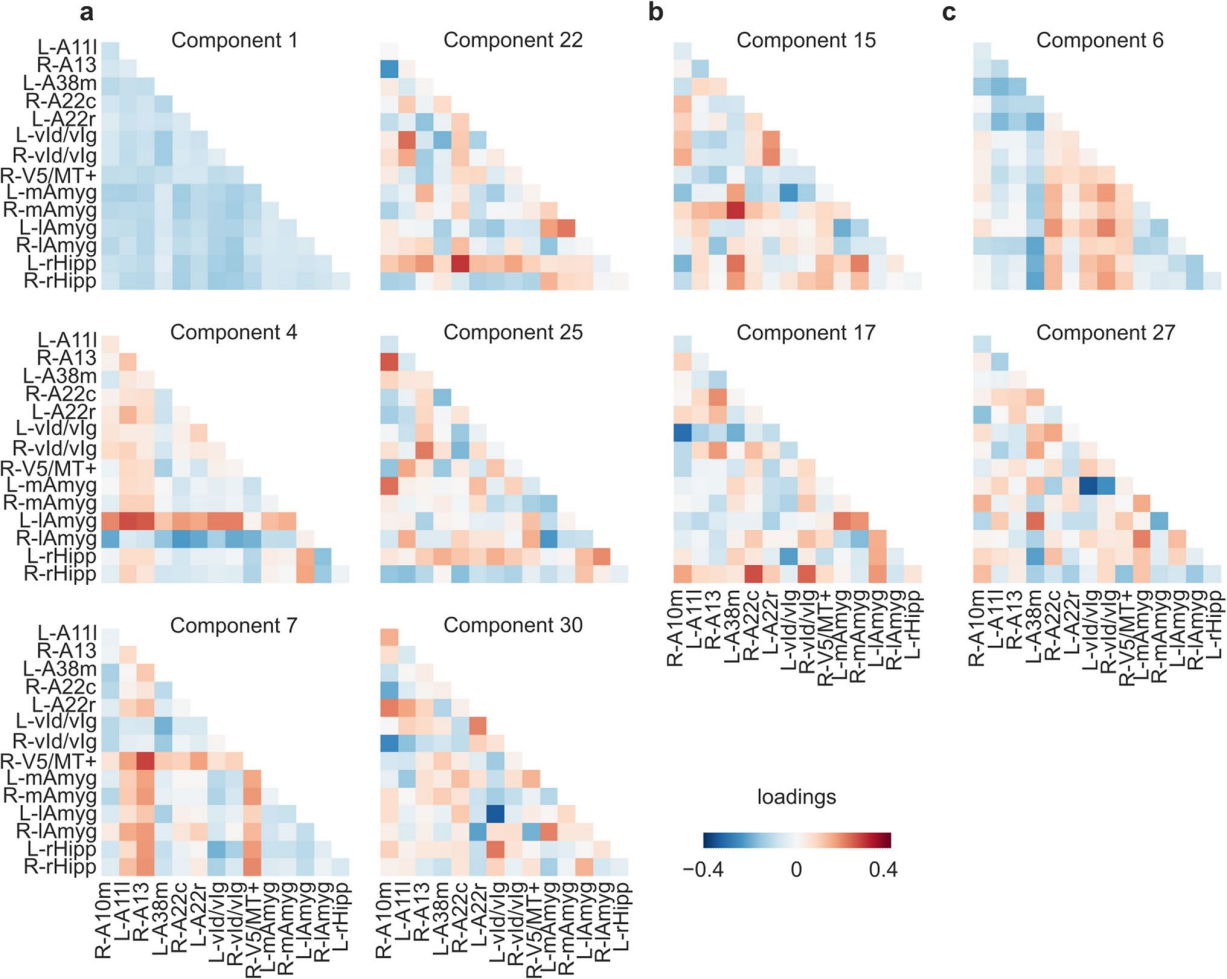
Detailed loadings of PCA coupling components. Respective loadings of the 105 raw connectivity measures to the 10 components retained by LASSO regularization (Table 4). (**a**) Components associated exclusively with positive affectivity. (**b**) Components associated with both positive and negative affectivity. (**c**) Components associated exclusively with negative affectivity

**Fig. 4:**
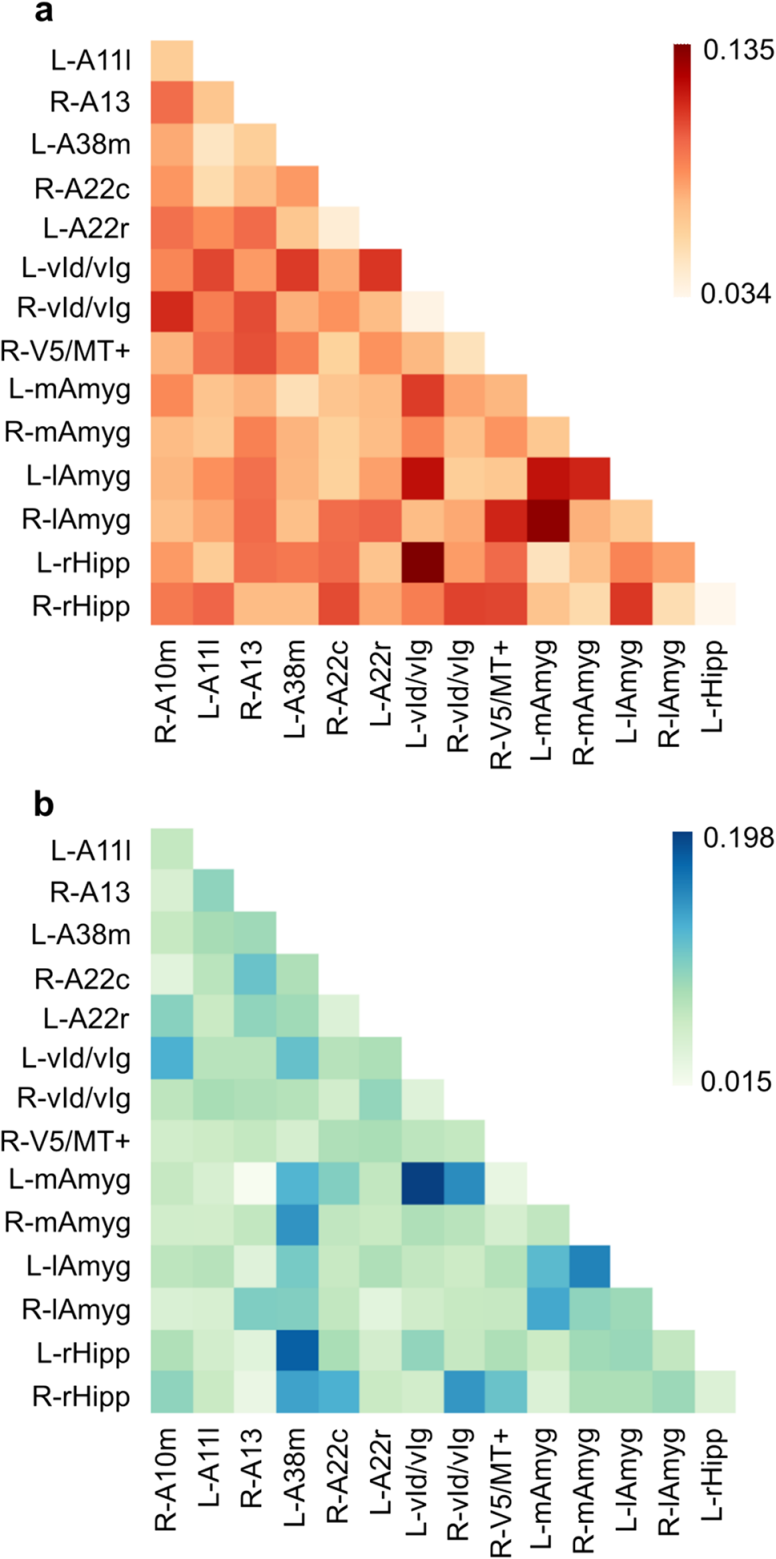
Summarized loadings of PCA coupling components. Averaged absolute PCA loadings are depicted across the coupling components retained by LASSO regularization, separately for positive (**a**) and negative (**b**) affectivity models, highlighting the higher contribution of the connectivity of the amygdala, hippocampus, and insula in explaining within-person variation in day-to-day affectivity

When fitting functional brain connectivity data (30 coupling components) from all available sessions, multivariate mixed-effect linear models explained 15.08% and 10.58% of within-person variation in positive and negative affectivity, respectively. LASSO regulation retained 8 (C1, C4, C7, C15, C17, C22, C25, C30) and 4 (C6, C15, C17, C27) coupling components to explain intra-individual variations in positive and negative global PANAS scores, respectively (Table 4). Two of these coupling components (C15 and C17) were shared by positive and negative affectivity models but with opposite signs. Permutation feature importance indicated that coupling components with the strongest contribution each accounted for > 1% of the within-person variation in affectivity (Table 4). Inspection of PCA loadings for the coupling components selected by LASSO regulation highlighted the stronger involvement of some functional brain connections over others (Fig. 3). Specifically, functional connectivity of the amygdala, hippocampus, and insula appeared to contribute more to explain within-person variation in affectivity than that of other brain areas, both for positive and negative affectivity (Fig. 4). When using cross-validation to test multivariate mixed-effect linear models on unseen data, a pronounced drop in the proportion of predicted within-person variation in affectivity was observed. On average across the five folds, models predicted 4.63% of the change in positive affectivity across sessions at the within-person level and 2.25% for negative affectivity. Mass univariate mixed-effect linear models that separately estimated associations between each of the 105 raw connectivity measures and positive or negative affectivity scores did not reveal any significant effect when correcting for multiple comparisons.

**Table 4:** Multivariate mixed-effect linear models. PCA coupling components retained by the LASSO regularization for models explaining positive and negative affectivity, respectively. Estimates indicate the magnitude and direction of each coupling component’s contribution. Notably, components C15 and C17 are shared between positive and negative affectivity models, although with opposite signs. The change in R^2^ (%) denotes the loss in the proportion of explained variation upon removing the influence of a given component, as revealed by permutation feature importance

Predictor (component)	Estimate	Std. Error	Change in R^2^ (%)
Positive affectivity			
C1	0.21	0.03	− 0.35
C4	0.35	0.06	− 0.34
C7	0.60	0.07	− 0.11
C15	0.85	0.09	− 1.07
C17	0.94	0.10	− 1.03
C22	− 1.25	0.11	− 1.33
C25	− 0.88	0.13	− 0.04
C30	1.30	0.14	− 0.12
Negative affectivity			
C6	− 0.51	0.06	− 1.31
C15	− 0.77	0.09	− 1.08
C17	− 0.63	0.10	− 0.68
C27	− 1.32	0.13	− 2.02

## Discussion

Expanding on previous resting-state fMRI work conducted in a single subject (Betzel et al., 2017; Mirchi et al., 2018), this study explored how changes in functional brain connectivity can account for within-person fluctuations in positive and negative affectivity over time in a small sample of healthy young individuals. Multivariate models leveraged functional connectivity of brain areas implicated in affective processes, in particular limbic areas, such as the amygdala, hippocampus, and insula, to explain a portion of intra-individual variability in both positive and negative affectivity. Such models could, to a much lower level, generalize and predict novel affect levels in the same subjects.

Brain regions whose functional connectivity predominantly contributed to explain within-person variations in affectivity were shared across positive and negative affect models. This finding aligns with previous research showing activation of the same valence-general regions in both extremes of hedonic valence (Guillory & Bujarski, 2014; Kober et al., 2008; Lindquist et al., 2016). Specifically, the bilateral lateral amygdala, left medial amygdala, and left ventral insula were the primary contributors to the model explaining positive affectivity. For negative affectivity, these regions were the bilateral medial amygdala, left temporal pole, right rostral hippocampus, and bilateral ventral insula. It is noteworthy that brain areas excluded from our selection based on meta-analytical criteria might have proved to be equally important; for example, the subgenual anterior cingulate cortex is a key brain region in mood disorders (Hamani et al., 2011; Lipsman et al., 2010; Price & Drevets, 2010). While the amygdala and insula are most traditionally associated with fear and disgust, respectively (Lindquist et al., 2012), they exhibit activation in both positive and negative affect (Guillory & Bujarski, 2014; Lindquist et al., 2012, 2016). More generally, the amygdala responds to motivationally salient stimuli, and the insula plays a role in the awareness of bodily sensations and interoceptions (Kober et al., 2008; Lindquist et al., 2012). Despite being only prominently featured in the negative model, the temporal pole is similarly recognized for its dual role in positive and negative emotions (Guillory & Bujarski, 2014; Olson et al., 2007). The rostral hippocampus, by contrast and consistently with this work, has been associated more with negative than positive valence, participating in hypothalamic–pituitary–adrenal axis regulation and serving as a therapeutic target in multiple affective disorders (Fanselow & Dong, 2010; Strange et al., 2014).

While some brain areas may play a valence-general role in affective processes, being associated with both positive and negative affectivity, it does not necessarily imply that they are similarly involved. Our findings highlight that two coupling components contribute to within-person variation for both positive and negative affectivity, exhibiting opposing associations—positively linked to positive affectivity and negatively correlated with negative affectivity. These results echo a previous meta-analysis of the neural correlates of affects that reported a bipolar pattern of activation in the ventromedial prefrontal cortex, which was activated during positive affects and deactivated during negative affects (Lindquist et al., 2016). In our study, the two components showing a bipolar relationship with fluctuating positive and negative affects were most strongly dependent on the connectivity of the medial amygdala, rostral hippocampus, ventral insula, and temporal lobe.

Associations between brain and behavior, as revealed by inter-individual level using cross-sectional data, have been shown to be far smaller than previously envisioned (Marek et al., 2022). Similarly, our findings indicate that only a modest part of the in-sample intra-individual variance in affectivity can be accounted for by longitudinal changes in functional brain connectivity (~ 10–15%), which is further diminished (up to 5% only) when assessing how our models generalize to new, out-of-sample data using cross-validation (Rosenberg & Finn, 2022; Yarkoni & Westfall, 2017). Comparing the predictive capability of our models with similar previous work is challenging given the nascent state of studying intra-individual effects in cognitive and clinical neuroimaging. Nevertheless, several avenues for improving the search of brain-behavior associations at the within-person level can be envisioned.

The strength of brain-behavior relationships depends on the reliability of measurements of both brain function and phenotypical assessments (Gell et al., 2023; Goodwin & Leech, 2006; Hedge et al., 2018; Nikolaidis et al., 2022; Tiego & Fornito, 2023). Reliability, indicating the consistency of measures in identical situations, is susceptible to fluctuations from random variability and measurement noise (Gell et al., 2023; Hedge et al., 2018). Reliability differs from validity, as stable measures may obscure meaningful variability (Finn & Rosenberg, 2021; Li et al., 2019; Noble et al., 2021; Zuo et al., 2019). The Day2day dataset includes a large amount of resting-state fMRI data per subject, approximately 4 h. Yet, only 5 min of data was obtained in each individual scanning session, falling short of the minimum of 30 min needed to ensure good intrasession reliability (Gordon et al., 2017; Gratton et al., 2018; Kraus et al., 2023; Laumann et al., 2015). Regarding phenotypical measures, internal affective states cannot be verified externally and depend on self-report measures (Barrett, 2004; Quigley et al., 2014; Wallbott & Scherer, 1989). The accuracy of self-reports can be affected by the subjects’ capacity for introspection (Haybron, 2007), ability to perceive and experience granularity in feelings (Barrett & Bliss-Moreau, 2009; Quigley et al., 2014), response biases (Paulhus, 2002; Wallbott & Scherer, 1989), the interpretation of questionnaire items (Zevon & Tellegen, 1982), and recall bias (Stone & Shiffman, 1994). Considering the latent traits of individual responses could inform more precisely on the specific state and reduce measurement noninvariance between participants (Tiego & Fornito, 2023). The use of multiple convergent phenotypic measurements also could inform on the true underlying state of the subject and increase reliability (Havron, 2022).

Several factors could enhance the ability to explain or predict the intra-individual variation in affectivity using functional brain connectivity patterns. First, the relatively low total number of sessions prompted us to focus our analyses on a very limited number of fine-grained brain areas, given the quadratic increase in the number of functional connections with each additional region. Inclusion of additional brain areas pertinent to affective processes, such as the subgenual anterior cingulate cortex (Hamani et al., 2011; Lipsman et al., 2010; Price & Drevets, 2010), is likely to contribute to improving models of affectivity dynamics. Second, the brain regions of interest were extracted in a common space for all subjects using a standardized brain atlas (Fan et al., 2016). While there are similarities in the functional topography of brain areas and networks across subjects, there also are notable idiosyncratic variations in individuals (Gratton et al., 2018; Kraus et al., 2023; Laumann et al., 2015; Mueller et al., 2013). It has been demonstrated that the use of personalized brain mapping to account for such spatial topography variations improves the prediction of emotion, personality, and cognitive ability at the between-person level. It is reasonable to postulate that employing this method would similarly lead to predictive improvements at the within-person level. Finally, and critically, our investigation of intra-individual dynamics was conducted at the group level only due to the small number of participants. However, substantial heterogeneity is expected across subjects, as reported by previous studies that investigated intra-individual effects linking cerebral function to mood and affect (Baucom et al., 2012; Kirkby et al., 2018; Sani et al., 2018).

Intra-individual research in affective neuroscience is in its early stages, yet it already yields promising findings that hold potential for enhanced patient monitoring and personalized treatments in psychiatry (Cornblath et al., 2019; Fernandes et al., 2017; Heller, 2016; Insel & Cuthbert, 2015; McMahon, 2014). As a notable example, it has been shown that a patient’s responses to direct electric stimulation can be state-dependent, either alleviating or worsening mood depending on the initial affective state (Scangos et al., 2021). Future work should aim to elucidate how brain-behavior relationships differ between trait-level and state-level analyses, effectively contrasting inter- and intra-individual variability, spanning various temporal scales, from minutes to days and months (Rosenberg et al., 2020). Moreover, beyond seeking concurrent associations, it will be interesting to evaluate the utility of brain measures in forecasting future moods. For instance, Lynch et al., (2023) found a correlation between current brain states and future levels of anhedonia (the absence of positive mood) in patients with depression. Critically, well-powered studies with large samples will be important not only to limit the effects of low reliability and maximize generalizability (Babyak, 2004; Gell et al., 2023; Yarkoni & Westfall, 2017; Ying, 2019) but also to enable the proper characterization of heterogeneity and the stratification of clinical populations.

## Data Availability

The Day2day dataset is available upon request to the authors of the original study.

## Code availability

The code used for brain imaging analyses is available online at https://github.com/pnplab/day2day_paper/.

## Declarations

### Open practices statement

The data used in this experiment are not publicly available, but they can be obtained by request to the authors of the original study. The experiment was not preregistered.

## Abbreviations

A10m: Medial area 10
A11l: Lateral Brodmann area 11
A13: Brodmann area 13
A22c: Caudal Brodmann area 22
A22r: Rostral Brodmann area 22
A38m: Medial Brodmann area 38
BOLD: Blood-oxygen-level-dependent
lAmyg: Lateral amygdala
LASSO: Least Absolute Shrinkage and Selection Operator
mAmyg: Medial amygdala
NA: Negative affectivity
PA: Positive affectivity
rHipp: Rostral hippocampus
ROI: Region Of Interest
FD: Framewise displacement
FDR: False discovery rate
fMRI: Functional Magnetic Resonance Imaging
vId/vIg: Ventral dysgranular and granular insula
V5/MT +: Visual area 5/Middle temporal visual area +
